# Cell-Mediated Immunity to NAGLU Transgene Following Intracerebral Gene Therapy in Children With Mucopolysaccharidosis Type IIIB Syndrome

**DOI:** 10.3389/fimmu.2021.655478

**Published:** 2021-05-10

**Authors:** Marie-Lise Gougeon, Béatrice Poirier-Beaudouin, Jérome Ausseil, Michel Zérah, Cécile Artaud, Jean-Michel Heard, Kumaran Deiva, Marc Tardieu

**Affiliations:** ^1^ Institut Pasteur, Innate Immunity and Viruses Unit, Infection and Epidemiology Department, Paris, France; ^2^ Service de Biochimie Institut Fédératif de Biologie, Centre Hospitalier Universitaire de Toulouse, Institut Toulousain des Maladies Infectieuses et Inflammatoires (Infinity), INSERM UMR1291 - CNRS UMR5051 - Université Toulouse III, Toulouse, France; ^3^ Pediatric Neurosurgery Department, Assistance Publique-Hôpitaux de Paris, Hôpital Necker; Institut Imagine, Université René Descartes; NeuroGenCell, Institut du cerveau et de la moelle, Paris, France; ^4^ Institut Pasteur, Centre for Translational Science, Clinical Core, Paris, France; ^5^ Institut Pasteur, Biotherapy and Neurodegenerative Diseases Unit, Neuroscience Department, INSERM U1115, Paris, France; ^6^ Pediatric Neurology Department, Assistance Publique-Hôpitaux de Paris, Hôpitaux Universitaires Paris-Saclay, Bicêtre Hospital and INSERM UMR 1184, Immunology of Viral Infections and Autoimmune Diseases, CEA, IDMIT, Le Kremlin-Bicêtre, France

**Keywords:** MPS IIIB, NAGLU, CNS, AAV, cellular immunity, T cells, cytokines

## Abstract

Mucopolysaccharidosis type IIIB syndrome (Sanfilippo disease) is a rare autosomic recessif disorder caused by mutations in the α-N-acetylglucosaminidase (NAGLU) gene coding for a lysosomal enzyme, leading to neurodegeneration and progressive deterioration of cognitive abilities in affected children. To supply the missing enzyme, several recent human gene therapy trials relied on the deposit of adeno-associated virus (AAV) vectors directly into the brain. We reported safety and efficacy of an intracerebral therapy in a phase 1/2 clinical trial (https://clinicaltrials.gov/ct2/show/NCT03300453), with a recombinant AAV serotype 2/5 (rAAV2/5) coding human NAGLU in four children with MPS IIIB syndrome receiving immunosuppression. It was reported that AAV-mediated gene therapies might elicit a strong host immune response resulting in decreased transgene expression. To address this issue, we performed a comprehensive analysis of cellular immunity and cytokine patterns generated against the therapeutic enzyme in the four treated children over 5.5 years of follow-up. We report the emergence of memory and polyfunctional CD4^+^ and CD8^+^ T lymphocytes sensitized to the transgene soon after the start of therapy, and appearing in peripheral blood in waves throughout the follow-up. However, this response had no apparent impact on CNS transgene expression, which remained stable 66 months after surgery, possibly a consequence of the long-term immunosuppressive treatment. We also report that gene therapy did not trigger neuroinflammation, evaluated through the expression of cytokines and chemokines in patients’ CSF. Milder disease progression in the youngest patient was found associated with low level and less differentiated circulating NAGLU-specific T cells, together with the lack of proinflammatory cytokines in the CSF. Findings in this study support a systematic and comprehensive immunomonitoring approach for understanding the impact immune reactions might have on treatment safety and efficacy of gene therapies.

**Graphical Abstract:**
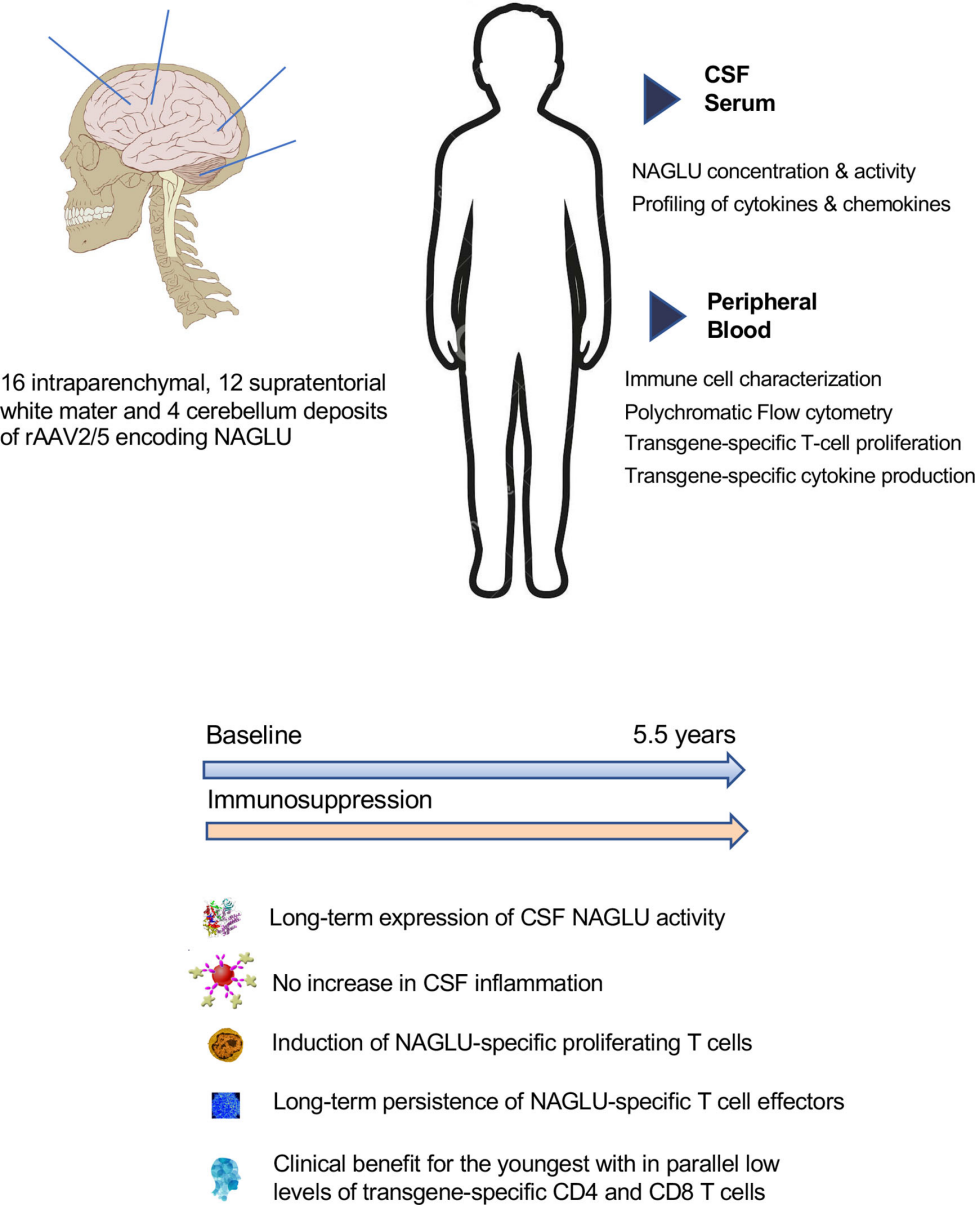


## Introduction

Mucopolysaccharidosis type IIIB syndrome (also known as Sanfilippo syndrome type B) is a rare autosomic recessif lysosomal storage disorder with predominant neurological manifestation in affected children. It is caused by mutations in the α-N-acetylglucosaminidase (NAGLU) gene, coding for a lysosomal enzyme required for the stepwise degradation of heparan sulfate glycosaminoglycans (GAGs). The accumulation of incompletely degraded GAGs in affected cells and extracellular spaces leads to cognitive retardation and further neurodegeneration of the central nervous system, leading to progressive deterioration of cognitive abilities before the age of 5 years, including language acquisition delay, cognitive delay and/or abnormal behavior, and premature death in the second decade (1–4). The challenge to treat MPS IIIB syndrome lies in the design of a therapy to supply the missing enzyme to the brain as early as possible after birth. Several recent human gene therapy trials for the treatment of neurodegenerative diseases relied on the deposit of adeno-associated virus (AAV) vectors directly into the brain (5–7). In preclinical studies in MPS IIIB mice (8, 9) and dogs (10, 11), beneficial biochemical and neurological effects with intracerebral gene therapy administered *via* a recombinant AAV vector encoding NAGLU were observed, associated with the release of therapeutic enzyme from transduced brain cells. These results led to the assessment of safety and efficacy of a novel intracerebral therapy in a phase 1/2 uncontrolled clinical trial, in which four children with MPSIIIB syndrome were enrolled to receive intraparenchymal deposits of a recombinant AAV vector serotype 2/5 (rAAV2/5) encoding human NAGLU combined with immunosuppression (ClinicalTrials.gov Identifier: NCT03300453). An intermediate report at 30 months concluded that treatment was well tolerated and induced sustained enzyme production in the brain. Good tolerance, sustained NAGLU production and milder disease in the patient treated at very early stage were confirmed after a 5.5 years follow-up (Deiva et al. Submitted to publication).

The success of gene therapy not only depends on the expression of the transgene but also on the ability to control the immune response directed against the vector particles and the transgenic protein used as a therapeutic agent. Early works in the field demonstrated that AAV-mediated gene therapies elicit a strong host immune response (12–14), resulting in safety concerns for the patient, decreased transgene expression, and decreased longevity of transgene expression (12, 13, 15, 16). Although the overall immunogenicity of AAV-based gene therapies is well characterized, immunogenicity from CNS-directed AAV delivery has not been widely investigated. Preclinical studies of CNS-directed AAV administration in non-human primates have reported AAV-specific circulating neutralizing antibodies and transgene-specific IFN-γ-producing T cells (17). Intraparenchymal delivery of AAV vector in the dog models of Hurler (18) and Sanfilippo (11) syndrome was reported to induce neuroinflammation including lymphocyte, plasma cells and macrophage infiltration into perivascular spaces and adjacent tissues. These observations support the administration of an immunosuppressive regimen in combination with CNS-targeting gene therapy (11). Immune responses in the CNS are common, despite its perception as a site of immune privilege, and microglia and astrocytes can engage in cross-talk with CNS-infiltrating T cells and other components of the innate immune system (19). The immune system of patients with null mutations perceives the AAV-derived transgene product as immunologically foreign and may develop severe T-cell responses against it.

To address this issue, we performed a functional analysis of T cell responses and cytokine patterns generated against the therapeutic enzyme NAGLU in children with MPSIIIB syndrome who received intracerebral rAAV2/5 encoding NAGLU plus immunosuppressive therapy. We saw the emergence of memory and effector CD4^+^ and CD8^+^ T lymphocytes sensitized to the therapeutic enzyme soon after the start of therapy, which induced sustained enzyme production in the brain. *Ex vivo*, these cells proliferated and produced type-1 cytokines when exposed to NAGLU antigenic epitopes. They are detected in peripheral blood in waves during the 5.5 years of follow-up in all four patients, and no acquired tolerance was observed at the end of the follow-up period. Moreover, a mild neuroinflammation is suggested by the levels of inflammatory cytokines and chemokines detected in patient’s CSF at several time points after gene therapy. This study is the first to report a comprehensive analysis of transgene-specific cellular immune response, conducted as part of a phase 1/2 clinical trial for intracerebral gene therapy in children with MPS III syndrome, with extensive follow-up of more than 5 years.

## Patients and Methods

### Study Design and Patients

Study design, regulatory aspects, inclusion/exclusion criteria, and neurosurgical procedures were previously described (20). Briefly, inclusion criteria were age 18 months to 4 years, clinical manifestations related to mucopolysaccharidosis type IIIB syndrome, and NAGLU activity in the blood less than 10% of that in unaffected children. Four children aged 20 (Patient 1), 26 (Patient 2), 30 (Patient 3) and 53 months (Patient 4) were enrolled between 17/09/2013 and 07/04/2014 in an uncontrolled, phase 1/2 clinical study. Main characteristics at diagnosis and inclusion were previously reported (20) (Deiva et al. submitted to publication). Surgery consisted in 16 intraparenchymal deposits of the gene transfer vector rAAV2/5 encoding human NAGLU. Each deposit was 60 μL and contained 2·4 × 10¹¹ vector genomes. Immunosuppression was started 14 days before surgery with 0.2 mg/Kg oral tacrolimus and 1200 mg/m2 oral mycophenolate mofetil (MMF) per day. The dose schedule for MMF was maintained for 6 weeks after surgery and tacrolimus doses were progressively reduced (trough concentrations in blood of 10-15 ng/ml for 3 months, 7-10 ng/ml from 3 months to 18 months 5-8 ng/ml from 18 months to 30 months, and 4 to 6 ng/ml from 30 months to 66 months). Tolerance, neurocognitive progression, NAGLU enzymatic activity in CSF, circulating T cells responding to NAGLU, and anti-AAV5 antibody were measured serially before and after surgery, as previously described (20).

### Biological Samples

The immunological assays were performed either on whole blood or PBMCs isolated from heparinized blood by Ficoll-Paque (Eurobio S.A., Montpellier) density gradient centrifugation. Assessments were done at baseline (BL), 1, 3, 6, 12, 30, 48 and 66 months after surgery. Serum and CSF were collected at the same timepoints and immediately frozen at -80°C.

### Lymphocyte Subsets and T Cell Phenotypic Characterization

Lymphocyte subset enumeration was performed by flowcytometry. 50µl of whole blood were mixed with anti-CD16 (clone 3G8, BD Pharmingen), -CD45-PerCP (clone 2D1, BD Biosciences), -CD19-PE-Cy7 (clone SJ25C1, BD Pharmingen), -CD4-V450 (clone RPA-T4, BD Horizon), -CD3-V500 (clone UCHT1, BD Horizon), -CD56-APC (clone B159, BD Pharmingen), and -CD8-APC-H7 (clone SK1, BD Pharmingen). Fifteen minutes incubation at room temperature was followed by 15 minutes incubation in 10 fold diluted BD FACS Lysing solution (Becton Dickinson). Stained cells were acquired on a CyAn Beckman Coulter and analysed with FlowJo software.

T cell phenotypic characterization on whole blood was determined with a nine-colour, 11 parameter staining cocktail composed of CD45-PerCP (clone 2D1, BD Biosciences), CD3-V500 (clone UCHT1, BD Horizon), CD4-V450 (clone RPA-T4, BD Horizon), CD8-APC-H7 (clone SK1 BD Pharmingen), CD45RA-PE-Cy7(BD Pharmingen), CCR7-PE (clone 3D12, BD Pharmingen), HLA-DR-FITC (clone G46-6, BD Pharmingen), CD69-APC (clone FN50, BD Biosciences), CD38-CF594 (clone HIT2, BD Horizon). The activation state of circulating lymphocytes was assessed by the expression of activation markers (CD38 and HLA-DR) on gated CD45^+^CD3^+^, CD45^+^CD3^+^CD4^+^ and CD45^+^CD3^+^CD8^+^ T cells. The proportions of naïve (N) (CD45RA^+^CCR7^+^), central memory (CM) (CD45RA^neg^CCR7^+^), effector memory (EM) CD45RA^neg^CCR7^neg^), and terminally differentiated (TD) (CD45RA^+^CCR7^neg^) T cells was also determined.

### NAGLU-Specific T Cell Proliferation Assay

We assessed T-cell responses against recombinant α-N-acetylglucosaminidase (rNAGLU) (R&D systems) and the synthetic NAGLU peptide (NAG peptide ab86400, Abcam) by measuring the proliferation of freshly isolated PBMC labelled with carboxyfluorescein succinimidyl ester (CFSE) (R&D Systems, Lille, France). As previously described (21), PBMCs were washed once with Phosphate buffered solution (PBS) (Life technologies, Saint Aubin, France) pre-heated at 37°C, and stained with 0.5µM CellTrace™ CFSE (CFSE) (Life technologies, Saint Aubin, France) for 15 minutes at 37°C. After washing with cold RPMI, PBMCs were stimulated for 4 days at 37°C in a 5% CO2 atmosphere with 0.5 µg/ml, 1µg/ml and 2 µg/ml of rNAGLU (R&D systems) or NAGLU peptide (77kDa form) at 0.5 µg/ml and 1µg/ml in the presence of 1µg/ml of CD28/CD49d mAbs. Staphylococcal enterotoxin B (SEB) (1 µg/ml) stimulation was used as a positive control and culture medium a negative control. At the end of the culture, cells were washed with PBS/10% BSA/0.1% NaN3 (PBA), stained with 7-AminoActinomycine-D (eBioscience) for dead cells staining, and co-stained with the following mAbs: CD3-V500 (clone UCHT1, BD Horizon), CD4-V450 (clone RPA-T4, BD Horizon), CD8-APC-H7 (clone SK1, BD Pharmingen), CCR7-PE (clone 3D12, BD Pharmingen), CD45RA-PE-Cy7 (clone H100, BD Pharmingen), CD69-APC (clone FN50, BD Pharmingen). Stained cells were then washed with PBA containing 20µg/ml Actinomycine-D (Sigma-Aldrich, Lyon France), fixed in 1% PFA solution, immediately acquired on a CyAn Beckman Coulter and analysed with FlowJo software (21). The frequency of NAGLU-specific proliferating CD4^+^ or CD8^+^ T cells was determined after removing the background in non-stimulated cultures.

### Whole Blood Intracellular Cytokine Staining (ICS) Assay

In order to determine the frequency of NAGLU-specific effector T cells, 700 µl of whole blood were stimulated for 6 hours at 37°C with NAGLU at 0.5 µg/ml, 1µg/ml or 2 µg/ml, or NAGLU peptide at 0.5 µg/ml or 1µg/ml in the presence of 1µg/ml CD28/CD49d mAbs and 10µg/ml Brefeldine A (Sigma-Aldrich, Lyon France). SEB (1 µg/ml) was used as a positive control. Blood samples were then treated with 10 fold diluted FACS Lysing solution (Becton Dickinson) and stored at -80°C until the ICS assay. Staining was performed on unfrozen blood samples, washed with PBA and permeabilized with BD FACS™ Permeabilizing Solution 2 (Becton Dickinson). The following mAbs were used: anti-CD3-V500 (clone UCHT1, BD Horizon), anti-CD4-APC (clone RPA-T4, BD Pharmingen), anti-CD8-APC-H7 (clone SK1, BD Pharmingen), anti-IFNγ-AF488 (clone B27, BD Pharmingen), anti-CCR7-PE (clone 3D12, BD Pharmingen), anti-CD45RA-PE-Cy7(BD Pharmingen), anti-IL2-PE-CF594 (clone 5344.111, BD Horizon), anti-CD69-PerCP (clone L78, BD Biosciences), and anti-TNFα-V450 (BD Horizon). Staining was done in PBA containing 0.05% saponin (Sigma-Aldrich, Lyon France), cells were fixed in 1% PFA solution and immediately acquired on CyAn Beckman Coulter. The frequency of T cells expressing CD69 and intracellular cytokines was determined with FlowJo software.

### Serum and CSF Cytokines and Chemokines Measurement

We used MAP technology for multiplexed quantification of cytokines in the CSF and plasma. At every time point, blood was drawn and lumbar puncture was performed. Plasma and aliquots of the CSF sample were centrifuged and the supernatant immediately frozen at −80°C. Samples were stored until use. Assessment of cytokine concentration on CSF and plasma samples was performed using a 27-plex kit (Human XL cytokine Premixed Magnetic Luminex Performance Assay Kit (R&D Systems, bio-techne) according to the manufacturer’s instructions. Analyzed cytokines included IL-1β, IL-1Ra, IL-2, IL-4, IL-5, IL-6, IL-7, IL-8, IL-9, IL-10, IL-12 p70, IL-13, IL-15, IL-17, Eotaxin, basic FGF, G-CSF, GM-CSF, IFN-γ, IP-10, MCP1, MIP-1α, MIP-1ß, PDGF, RANTES, TNF-α and VEGF. In brief, 50 µl of standard, serum or CSF were incubated with antibody-linked beads for 2 h. Samples were then washed three times with wash solution, and incubated for 1 h with biotinylated secondary antibodies. A final incubation of 30 min with streptavidin-PE preceded the acquisition on the Bioplex 200 (Biorad). At least 100 events were acquired for each analyte. Values below the standard curves were replaced by the lowest values of the concentrations measured. In order to enable an optimal comparability between different patients and timepoints, all CSF and plasma samples were measured at the same time on a same multiplex plate.

### NAGLU Concentration and Catalytic Activity

Protein, glucose concentrations, cell counts and NAGLU catalytic activity were determined in CSF samples concentrated six times and in non-concentrated plasma samples, as previously described (20).Briefly, proteins in 500 μl of CSF were titrated and concentrated 6 times and the NAGLU catalytic activity was measured in 50 concentrated μl of CSF by adding fluorescent 4-methylumbelliferyl-2-acetamido-2-deoxy-α-D-glycopyranoside for 18 hours at 37°C followed by optic deviation lecture (excitation 355nm, emission 440nm). As a control of sample quality, the activity of α-L-iduronidase, which is the deficient enzyme in MPSI syndrome, was simultaneously measured and was found to be normal in patients with MPSIIIB syndrome compared with the normal value in unaffected children. Measurements were made serially before and after surgery in CSF samples from enrolled patients and compared with mean values in CSF or plasma samples obtained in the same hospitals from unaffected children with no CSF abnormalities. Results are expressed in nmol h–¹ mL–¹ of CSF, the limit of quantification being <5 nmol h–¹ mL–¹.

### Statistical Analysis

Statistical analysis was performed using GraphPad Prism version 9 (GraphPad software, San Diego, CA). Nonparametric measures of associations were used, including the Mann–Whitney *U*‐test, the Wilcoxon signed rank test, linear regression and Spearman rank correlation. *P <*0.05 was considered significant.  

## Results

Intracerebral rAVV2/5 gene therapy in the four enrolled children with MPSIIIB syndrome was well tolerated. At intermediate analysis 30 months after surgery, neurocognitive progression was improved in all patients, with the youngest patient (P1) having function close to that in healthy children (20). At final analysis 66 months after surgery, significantly higher cognitive performances were observed in P1, at each time point as compared with the natural history of the disease. In contrast, cognitive benefit was not observed anymore in the three oldest patients (P2, P3 and P4), with disease course similar to the reference population (Deiva et al. submitted to publication). Brain imaging was normal in P1 without brain atrophy, whereas brain atrophy was present in patients 2, 3 and 4 (Deiva et al. submitted to publication). NAGLU activity was not detected in any patient’s CSF at inclusion. The therapy induced sustained enzyme production in the brain of all patients, and NAGLU activity levels measured in patients’ CSF were equivalent in the four enrolled patients and corresponded to an average of 18.2% of the levels detected in unaffected children (20) (Deiva et al. submitted to publication). Vector genomes were detected in blood for 2 days after surgery, and NAGLU activity, slightly above the level of quantification, was transiently detected in plasma 1 month after treatment in P1, P2 and P3 (20). This suggests that particles deposited in brain tissue transited into the systemic circulation. Considering that neutralizing antibodies against adeno-associated viral vector serotype 5 (AAV5) were not detected in serum samples collected at inclusion or during follow-up (20), we thought important to perform a comprehensive analysis of cellular immune response.

### Time Course Distribution of Lymphocyte Subsets

**Figure 1:**
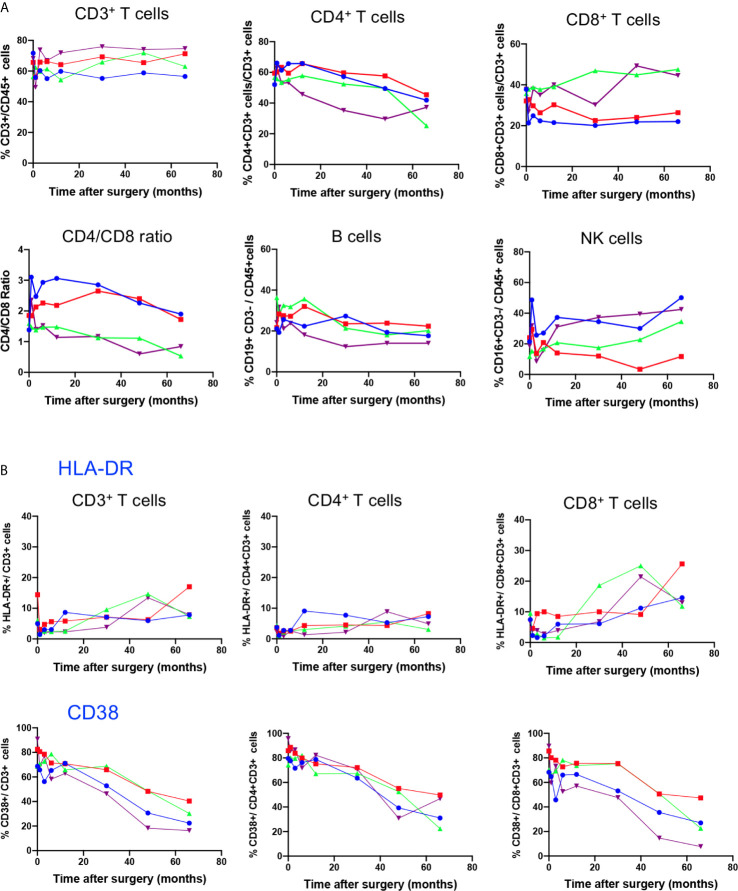
Time course distribution of lymphocyte subsets and expression of activation markers. **(A)** Lymphocyte subset enumeration was performed at indicated time points on whole blood by polychromatic flowcytometry. Cell subsets were characterized on gated CD45^+^ cells through the expression of CD3 (CD3^+^ T cells), coexpression of CD3 and CD4 (CD4^+^ T cells) or CD3 and CD8 (CD8+ T cells), CD3^neg^ CD19^+^ (B cells) and CD3^neg^ CD16^+^ (NK cells). Individual values of each patient (P1, P2, P3, P4) are shown. **(B)** The expression of the activation markers HLA-DR and CD38 on indicated T cell subsets was assessed on whole blood by polychromatic flowcytometry at indicated time points after gene therapy. Individual values of each patient (P1, P2, P3, P4) are shown.

To study the possible impact of gene therapy on the distribution of peripheral lymphocyte subsets during the 5.5 years post-therapy, we first measured the frequency of CD3^+^, CD3^+^CD4^+^ and CD3^+^CD8^+^ T cell subsets, B cells and NK cells in whole blood for each patient at BL, M1, M3, M6, M12, M30, M48, and M66 after surgery. Time course study depicted in **Figure 1A** shows stable frequency of CD3^+^T cells and B cells (CD19^+^CD3^neg^) in all 4 patients. However, with regard to T cell subsets, a progressive decrease in the frequency of CD3^+^ CD4^+^ T cells was detected in all patients throughout the study, and an increase in the frequency of CD3^+^CD8^+^ T cells was observed in P3 and P4, leading to a progressive decrease in CD4/CD8 ratio. This evolution is not different from that in healthy children who experience a natural decrease in the frequency of CD4^+^ T cells and increase in the frequency of CD8^+^ T cells during the first 6 years of life, as shown by Tosato et al. (22), who provided reference values for lymphocyte subsets in childhood. The frequency of NK cells (CD45^+^CD3^neg^CD16^+^) dropped at M1 in all four patients and progressively returned to BL, except for P2 (**Figure 1A**). Overall, this time course study shows that intracerebral gene therapy, associated with the chosen immunosuppressive treatment, had no serious impact on peripheral lymphocyte subset distribution.

### Impact of Gene Therapy on T Cell Activation and Memory Subset Distribution

To test whether gene therapy might trigger transient chronic activation of the immune system, the expression of HLA-DR and CD38 activation markers was analysed in T cell compartments (**Figure 1B**). Baseline expression of HLA-DR was low in CD3^+^ T cells, at similar level to those of age-matched healthy children (22). The time course study showed a slight increase in HLA-DR expression in CD3^+^ T cells, reflecting increased expression of this activation marker in the CD8^+^ T cell compartment from BL to M66 (P1: 7 to 14%; P2: 4 to 26%; P3: 9 to 12%; and P4: 6.6 to 13%). In contrast, HLA-DR expression in the CD4 T cell compartment remained low and stable (**Figure 1B**). CD38 was expressed in an important fraction of CD3^+^T cells (68% to 91%), CD4^+^ T cells (74% to 96%) and CD8^+^ T cells (68 to 90%) at BL, as reported for age-matched healthy children (22), and it decreased progressively during the time course study (ranging from 16 to 40% of CD3^+^ T cells, 22 to 50% of CD4^+^ T cells and 8 to 27% of CD8^+^ T cells at M66), according to age-dependent changes reported for healthy children (22).

**Figure 2:**
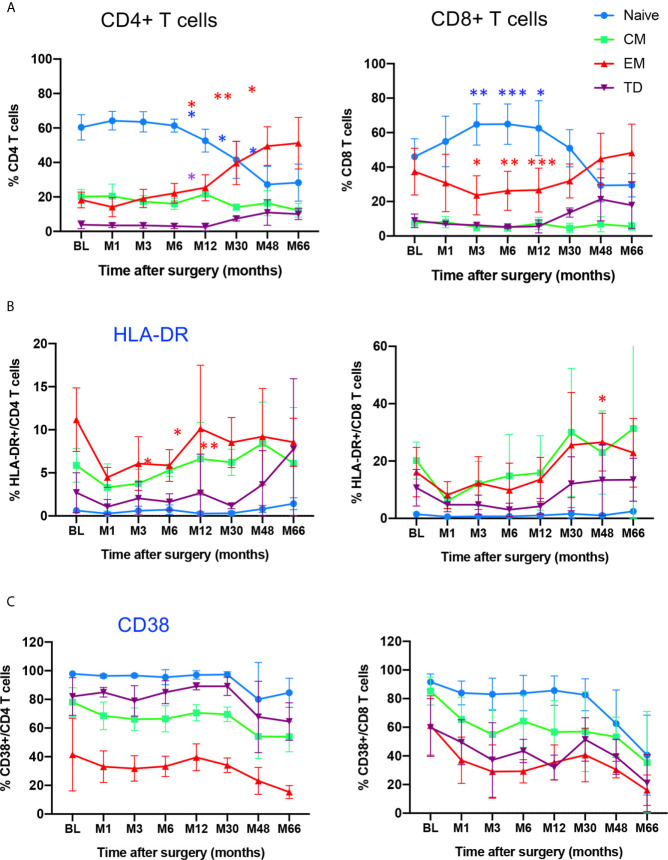
Time course distribution and activation state of memory/effector CD4^+^ and CD8^+^ T cells. **(A)** Whole blood distribution of naïve/memory/effector subsets in CD4^+^ and CD8^+^ T cells. The percentage of cell subsets within CD4^+^ and CD8^+^ T cells are shown at indicated time points. **(B)** HLA-DR expression in indicated subsets within CD4^+^ and CD8^+^ T cells. **(C)** CD38 expression in indicated subsets within CD4^+^ and CD8^+^ T cells. N, naïve, CM, Central Memory; EM, Effector Memory; TD, Terminally Differentiated, identified through CD45RA/CCR7 expression. Mean value ± SD are shown. Statistical comparisons for each time point vs baseline are indicated. *0.01 <p< 0.05, **0.001<p< 0.01, ***0.0001<p<0.001.

The combined use of CD45RA and CCR7 markers allowed to identify four phenotypically and functionally distinct populations in both CD4 and CD8 compartments: naive (N: CD45RA^+^CCR7^+^), central memory (CM: CD45RA^-^CCR7^+^), effector memory (EM: CD45RA^-^CCR7^-^) and terminally differentiated cells (TD: CD45RA^+^, CCR7^-^) cells. **Figure 2A** shows the time course evolution of these subsets following gene therapy. At BL, N T cells were the most prominent population in the CD4 compartment, accounting for 60.3 ± 7% (mean ± SD) of total CD4 T cells, memory and effector subsets accounting for 20.2 ± 4% (CM), 18.2 ± 4% (EM), 3.9 ± 2% (TD) of CD4^+^ T cells. Following gene therapy, a progressive increase in the frequency of EM was detected, which was statitically significant from M30, reaching at M66 51 ± 14% of CD4^+^ T cells. Similarly, although less pronounced, the frequency of TD cells increased significantly à M30, reaching 10 ± 3% of CD4^+^ T cells at M66. In parallel, a drop in both N and CM CD4^+^ T cells was observed, from 60.3 ± 7% at BL to 28 ± 10% at M66 for N CD4^+^ T cells, and from 20.2 ± 4% at BL to 12.2 ± 3.9% at M66 for CM CD4 T cells. Similar kinetics were observed for the CD8^+^ T cell compartment, N CD8^+^ T cells dropping from 46.1 ± 10% at BL to 29.5 ± 6.8% at M66, EM and TD CD8^+^ T cells rising from 37.3 ± 13% to 48 ± 16% and 8.9 ± 3.8% to 18 ± 13%, respectively (**Figure 2A**). Altogether, the kinetics of naïve and memory CD4 and CD8 compartments over the study period are compatible with an immune response to the viral vector used for gene therapy. Accordingly, analysis of the activation state of the memory/effector subsets shows a progressive increase in the expression of HLA-DR by CM, EM and TD CD4^+^ and CD8^+^ subsets while N cells did not express this activation marker (**Figure 2B**). With regard to CD38 expression by naïve and memory CD4 and CD8 subsets, a drop was detected for all naïve/memory subsets (**Figure 2C**), as observed for the whole population (**Figure 2A**), most probably due to age-dependent changes (22).

### Proliferative T Cell Response to NAGLU

**Figure 3:**
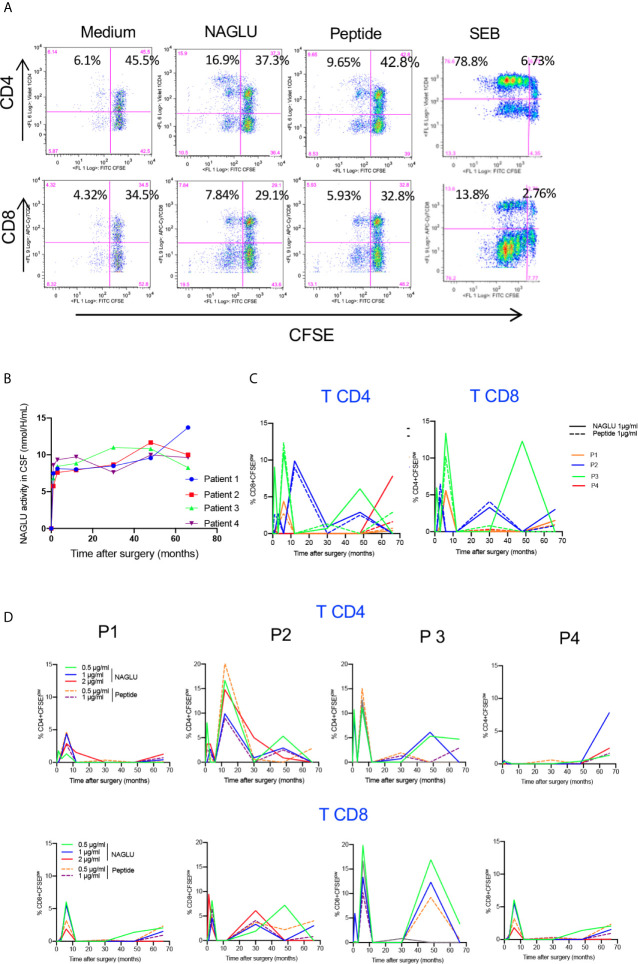
*Ex-vivo NAGLU-specific proliferative response.* **(A)** Representative flow charts showing the proliferation of CD4^+^ and CD8^+^ T cells on gated CD3^+^ T cells in response to NAGLU (1 μg/ml), NAGLU peptide (1 μg/ml), SEB (1 μg/ml), or unstimulated (medium), assessed with the CSFE assay. Freshly isolated PBMCs were stained with CFSE and stimulated for 4 days with the indicated antigens. Percentage of proliferating CFSE^low^ cells is indicated in the quadrants. **(B)** Time course evolution of NAGLU catalytic activity, assessed in concentrated CSF as described in “*Patients and Methods*”. **(C)** The *ex-vivo* detection of NAGLU-specific proliferating CD4^+^ and CD8^+^ T cells was assessed at baseline (BL) and 1, 3, 6, 12, 30, 48 and 66 months after surgery using the CFSE assay on freshly isolated PBMCs, stimulated for 4 days with the indicated concentrations of NAGLU or the peptide. The percentage of CFSE^low^ proliferating cells within CD4^+^ and CD8^+^ T cells, obtained after removing the background in non-stimulated cultures, is indicated for each patient over the longitudinal follow-up. **(D)** Overlay of patients’ CD4^+^ and CD8^+^ T cell proliferating responses to NAGLU and peptide at 1 μg/ml.

Since NAGLU is a neoantigen, it was important to identify a possible *in vivo* T cell sensitization following gene therapy. To do so, we first tested the *ex-vivo* T cell proliferative response to NAGLU using CFSE, a fluorochrome whose per cell fluorescent intensity halves with each round of cell proliferation (23). Freshly isolated PBMCs were labelled with CFSE and stimulated for 4 days with rNAGLU or NAGLU peptide. SEB was used as a positive control. Proliferating T cells were identified as CFSE^low^ cells excluding 7-AAD (living cells). **Figure 3A** shows representative dot plots of CFSE staining on gated CD3^+^ T cells. Both rNAGLU and NAGLU peptide were able to induce an increased percentage of CFSE^low^ CD4^+^ and CFSE^low^ CD8^+^ T cells compared to non-stimulated cells. As expected, SEB was a very potent inducer of CD4^+^ T cell proliferation. NAGLU catalytic activity, measured in CSF samples throughout the study, was not detected at inclusion but it rapidly reached a plateau from the 1^st^ month and did not vary until the end of the follow-up (**Figure 3B**). However, this activity was 15–20% of that in unaffected children (20). NAGLU activity was also undetectable in patients’ plasma at inclusion, but was slightly above the level of quantification 1 month after treatment and then undetectable over follow-up (20). *Ex-vivo* NAGLU-specific T cell proliferation was not detected at inclusion for any of the patients. Nevertheless, from M1 to M66 after surgery, NAGLU-specific proliferating CD4^+^ and CD8^+^ T cells were detected at all concentrations of NAGLU and peptide (**Figure 3C**). Strikingly, their detection was transient, appearing in waves over the follow-up for the four patients (**Figure 3C**). The overlaying of CFSE^low^ CD4^+^ and CD8^+^T cells detected during the 5.5 years of follow-up shows that the kinetics of appeareance and disappearance of these cells are comparable in the four patients (**Figure 3D**). They were still detected at the end of the follow-up in both T cell subsets, suggesting that immunological tolerance to NAGLU was not acquired 5.5 years after surgery.

### Kinetics of Cytokine-Producing T Cells in Response to NAGLU

**Figure 4:**
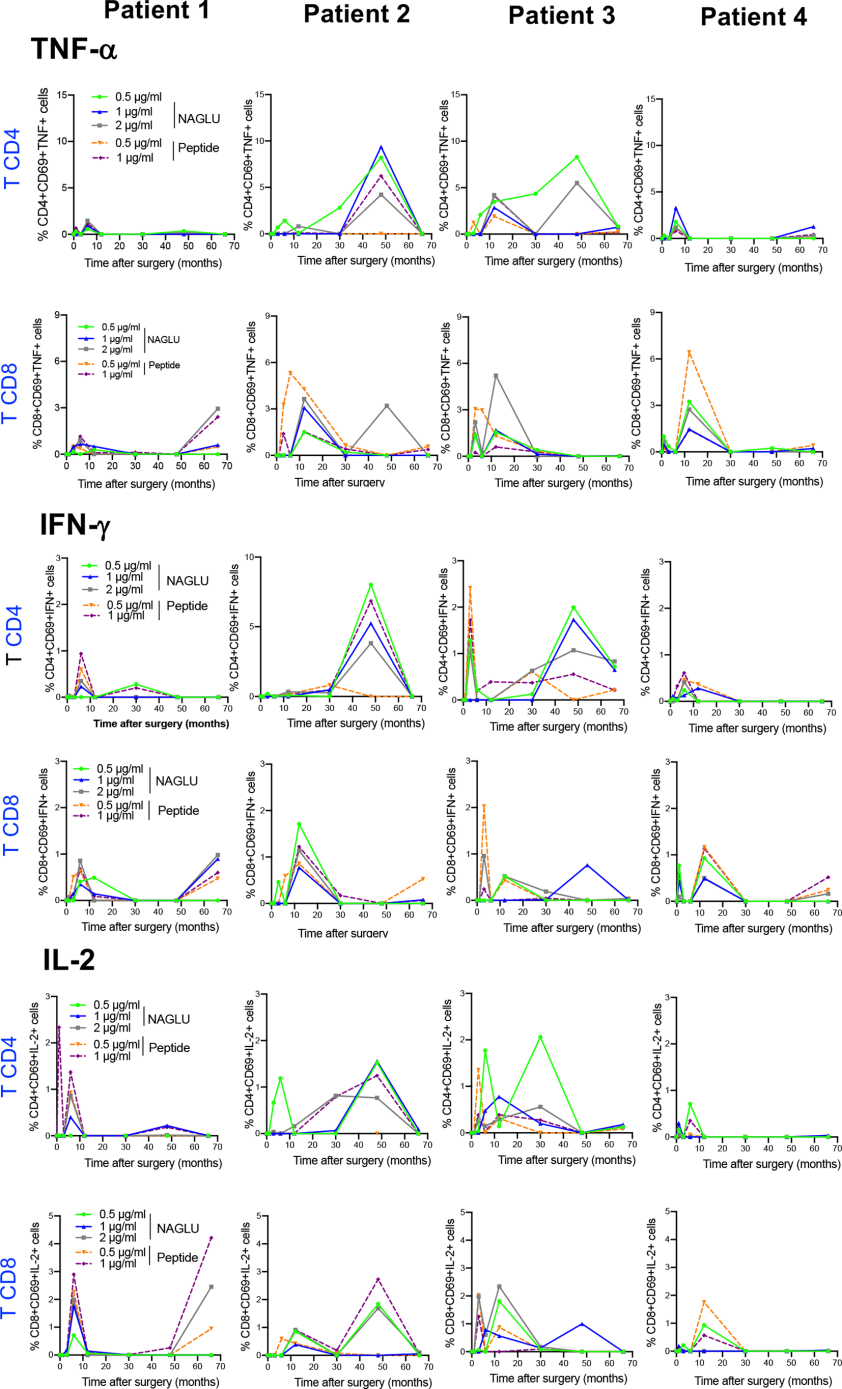
Time course evolution of ex-vivo NAGLU-specific cytokine expressing CD4^+^ and CD8^+^ T cells. Whole blood was stimulated for 6 h at indicated concentrations of NAGLU or the peptide, and specific CD4^+^ and CD8^+^ T-cell responses were measured using an intracellular cytokine staining assay. For each patient, the percentage of NAGLU-specific CD69^+^ CD4^+^ or CD8^+^ T cells expressing TNF-α, IFN-γ or IL-2 is plotted at baseline (BL) and 1, 3, 6, 12, 30, 48 and 66 months after surgery.

**Figure 5:**
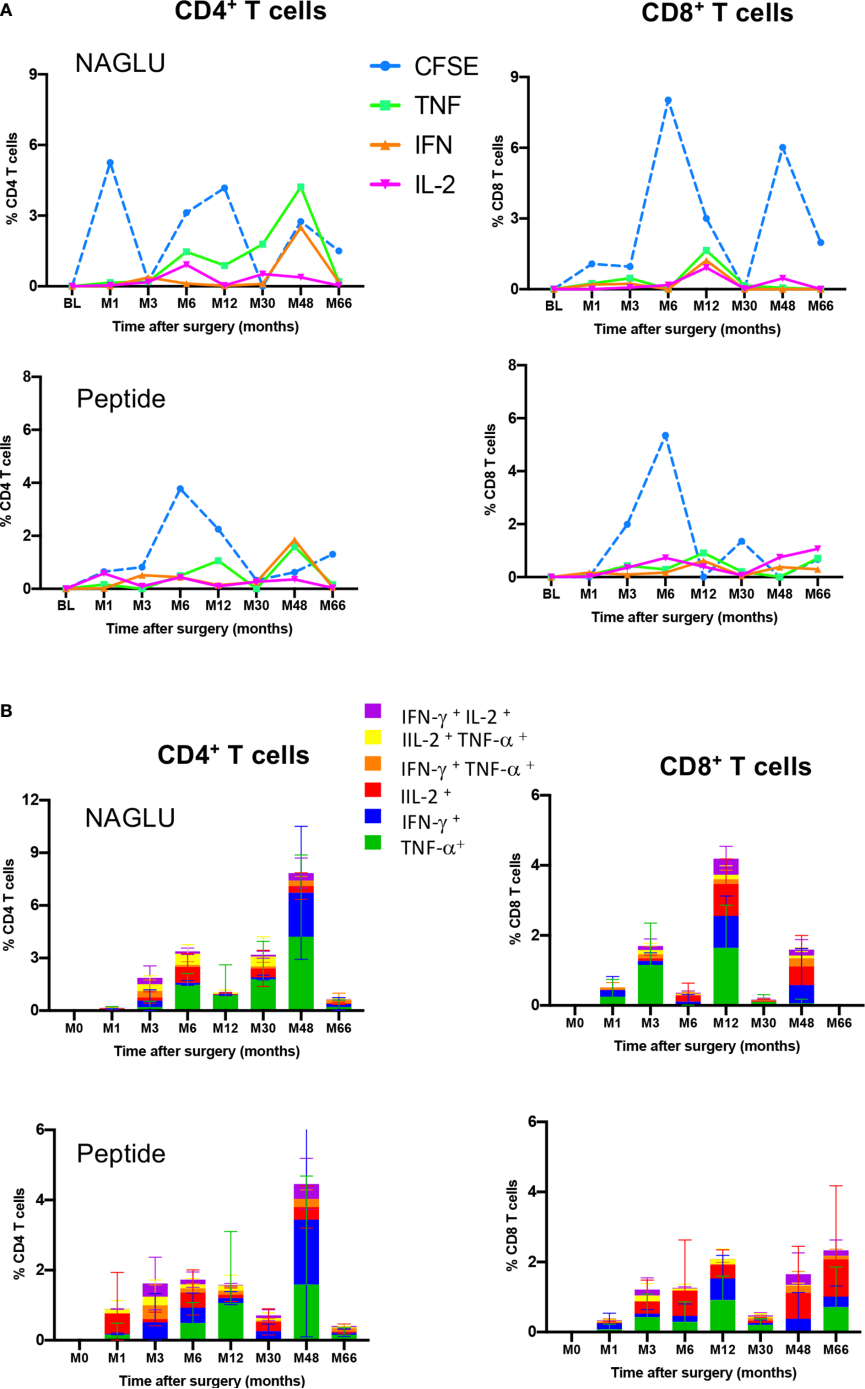
Functional profiles of NAGLU-specific circulating CD4^+^ and CD8^+^ T cells. **(A)** Overlay of *ex-vivo* proliferation and TNF-α, IFN-γ or IL-2 expression by CD4^+^ and CD8^+^ T cells after exposure of patients’ PBMCs to NAGLU (0.5 μg/ml) or peptide (1 μg/ml) measured at baseline (BL) and 1, 3, 6, 12, 30, 48 and 66 months after surgery. Mean percentage of NAGLU-specific CFSE^low^ cells or cytokine-expressing cells within CD4^+^ or CD8^+^ T cells is plotted for each time point. **(B)** CD4^+^ and CD8^+^ T-cell polyfunctionality analysis after whole blood stimulation for 6 h with NAGLU (0.5 μg/ml) or peptide (1 μg/ml). Bar charts represent the proportion of NAGLU-specific CD4^+^ or CD8^+^ T-cells displaying each particular cytokine or combination of cytokines at indicated time points of the follow-up. Data are presented as mean ± SD of specific responses from the four patients.

We completed cellular immunity study using the intracellular cytokine assay (ICS) on whole blood. Cytokine-expressing T cells responsive to NAGLU or the peptide were identified by the co-expression of CD69 and at least one of the type-1 cytokines TNF-α, IFN-γ or IL-2. NAGLU-specific cytokine-producing CD4^+^ T cells were not detected at inclusion. Upon *ex-vivo* exposure to NAGLU, CD4^+^ T cells producing TNF-α, IFN-γ or IL-2 became detectable at 3 months post surgery (**Figure 4**). However, as observed for NAGLU-specific proliferating T cells, cytokine-expressing T cells appeared in waves all along the kinetics study. Although low in frequency at the end of the follow-up at M66, cytokine-producing CD4^+^ T cells persisted. Similar observations were made for the CD8^+^ T cell compartment (**Figure 4**). The *ex-vivo* detection of proliferating and cytokine T cell responses to NAGLU reflects the *in vivo* induction of memory cells to the transgene, and their overlay (**Figure 5A**) indicates similar kinetics in both CD4 and CD8 compartments, but with a delay of at least 3 months between proliferative and cytokine responses.

### Polyfunctionality of Circulating NAGLU-Specific CD4^+^ and CD8^+^ T Cells

T-cell polyfunctionality has been largely considered an important metric reflecting the quality of the T-cell response (24). We assessed the functional pattern of CD4 and CD8 T cell compartments considering cells that they were able to mediate either one, two, or three cytokines in response to NAGLU (0.5 μg/ml) or the peptide (1 μg/ml). The total response was defined as the sum of all cells positive for at least one cytokine, and it provides a view of the overall frequency of responding cells. **Figure 5B** shows T cell functionality according to the number of cytokines produced, and responses were color-coded (mean ± SD). The functional profile displayed by CD4^+^ T cells in response to NAGLU was characterized by a prevailing representation of TNF-α-producing cells (green) over IFN-γ- (blue) or IL-2- (red) during the 30 month-period post-surgery. At M48, 85% of responsive cells expressed either TNF-α or IFN-γ, while the frequency of CD4^+^ T cells expressing 2 cytokines was 9%. Very similar functional profiles were obtained for CD4^+^ T cell response to the peptide (**Figure 5B**). Regarding the CD8^+^ T cell compartment, the functional pattern of NAGLU-reactive T cells followed a different kinetics. The relative distribution of cells expressing single TNF-α, IFN-γ, IL-2 or two cytokines, observed at M48 in CD4^+^ T cells, was detected earlier (at M12) in CD8^+^ T cells. At M48, the functional pattern of NAGLU-reactive CD8^+^ T cells was dominated by cells expressing lFN-γ (blue) or IL-2 (red) or both cytokines simultaneously (purple). It should be noted that no T cells producing 3 cytokines were detected at any time point, in contrast to T cell responses specific for persistent pathogens (25–27).

### Baseline CSF and Plasma Cytokine/Chemokine Profiles

**Figure 6:**
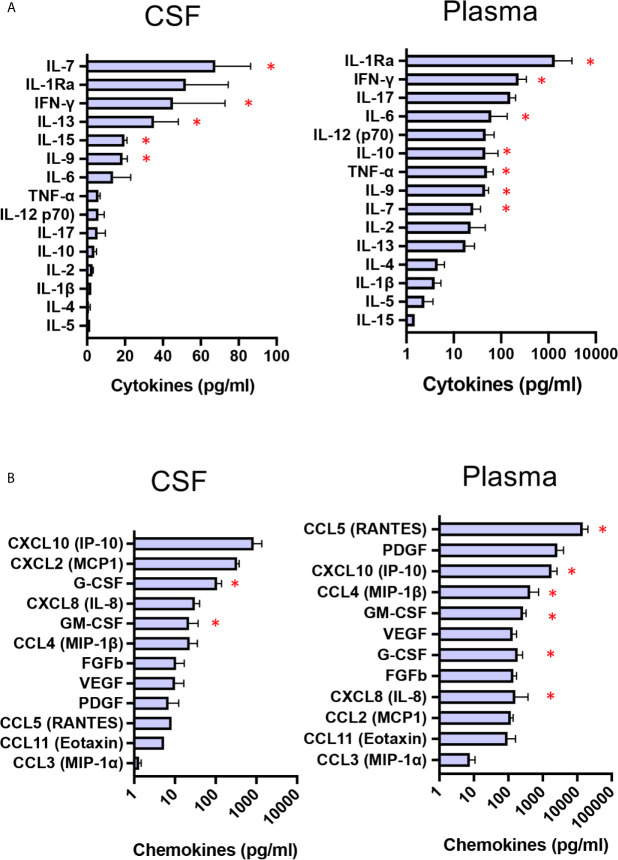
Baseline CSF and plasma cytokine/chemokine profiles. Boxplot diagrams of cytokine **(A)** and chemokine **(B)** concentrations in CSF and plasma from the four patients at baseline, assessed by multianalyte profiling of 27 cytokines and chemokines. Red star highlights cytokines or chemokines for which the values are markedly higher than those for healthy children (29). Data are presented as mean ± SD of cytokine/chemokine concentrations.

CNS-targeted delivery of AAV was reported to induce neuroinflammation (28), and preclinical studies using dog models of Sanfilippo with intraparenchymal delivery of AAV vector reported intense inflammation in the thalamus and macrophage infiltration into perivascular spaces (11). Pathology was not corrected unless immunosuppressant regimen was delivered to preventing inflammatory response and allowing disease correction in the brain of treated MPSIIIB dogs (11). Consequently, immunosuppression was started 14 days before surgery and progressively reduced from M3 to the end of the follow-up at M66. Taking advantage of multianalyte profiling (MAP), we have performed an analysis of 27 cytokines and chemokine in CSF and plasma from the four patients at baseline, with the aim to evaluate neuroinflammation and peripheral inflammation. Whereas 9 out of 15 cytokines i.e. IL-5, IL-4, IL-1ß, IL-2, IL-10, IL-17, IL-12,TNF-α and IL-6, in CSF were detected at very low concentrations and at levels comparable to that of healthy children (29), 6 cytokines i.e. IL-9, IL-15, IL-13, IFN-γ, and IL-7, barely detectable in CSF from heathy children (29)were found at high concentrations in patients’s CSF (**Figure 6**, red star). Regarding CSF chemokines and growth factors, all of them, except GM-CSF and G-CSF, had levels comparable to those of healthy children (29). Mean concentrations of GM-CSF and G-CSF were 22 and 108 pg/ml respectivey, whereas these growth factors are barely detected in CSF from healthy children (29) (**Figure 6**). Cytokine pattern in patients’ plasma at baseline was characterized by increased levels of infammatory cytokines, i.e. IL-7, IL-9, TNF-α, IL-10, IL-6, IFN-γ and IL-1 Ra, compared to healthy children (29) (**Figure 6**). Chemokine pattern in patients’ plasma was characterized by significant levels of IL-8, G-CSF and MIP-1ß (mean 158 pg/ml, 183 pg/ml, 431 pg/ml respectively), while these chemokines are not detectable in plasma from healthy children (29). GM-CSF (mean concentration 267 pg/ml, around 3 times more than healthy children), IP-10 (mean concentration 1800 pg/ml, around 20 times more than healthy children, and RANTES (mean concentration 14 430 pg/ml, around 14 times more than healthy children (29) were also highly increased. Overall, these results indicate a status of neuroinflammation, as well as peripheral inflammation, in the enrolled patients at baseline.

### Impact of Gene Therapy on Neuroinflammation

**Figure 7:**
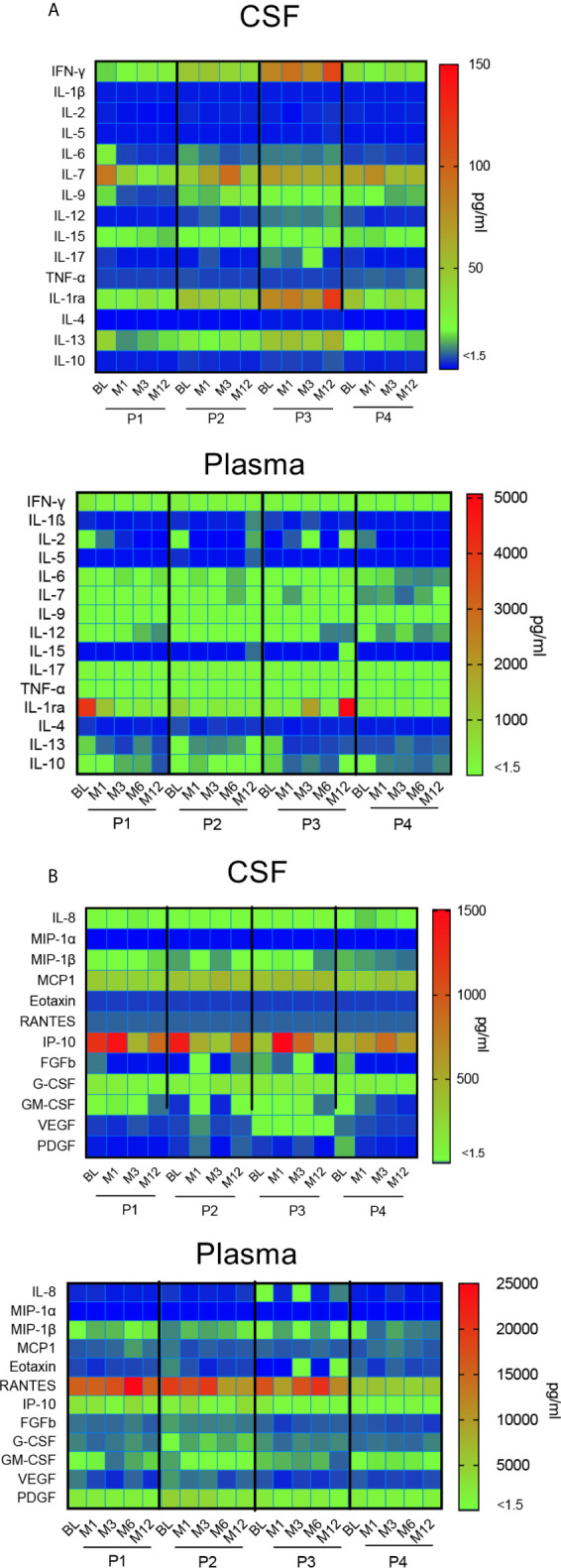
Impact of gene therapy on neuroinflammation. Heat map representing the broad array of cytokines **(A)** and chemokines **(B)** assessed by multianalyte profiling of 27 metabolites in patients’ CSF and plasma, at indicated time-points. The colored scale bar shows the range of concentrations expressed in pg/ml.

A time course analysis of cytokines and chemokines in CSF and plasma was performed at each time-point from BL to M12. Data are presented in a heat map (**Figure 7**). In CSF, the great majority of cytokines remained stable during the first year after gene therapy, except two inflammatory cytokines, i.e. IFN-γ and IL1Ra (**Figure 7A**). This mainly concerns P3, whose profile is characterized by the progressive increase of these 2 inflammatory cytokines, albeit stable in the three other patients. CSF IL-7 levels were slightly fluctuating in P2 and P4. With regard to CSF chemokines, all remained stable during the first year after gene therapy, except IP-10. Different kinetics were observed according to the patients, with a gradual decrease in P1, and a progressive increase in P3 (**Figure 7B**). Plasma cytokines remained stable for all four patients, with one exception for IL-1Ra, which strongly increased in P3. All chemokine levels remained stable in all patients.

Overall, this multiplex analysis of cytokines and chemokines in CSF suggest that intracerebral gene therapy did not trigger neuroinflammation during the first year after surgery in 3 out of 4 immunosuppressed patients.

## Discussion

AAVs can trigger innate or adaptive immune responses against the vector and the transgene (16, 30–32) and the intensity of the immune response depends on the route of delivery and the dose of vector that is delivered (33). CNS-directed delivery allows to reduce the overall immune response because it requires lower doses of vector to reach clinically relevant transgene expression in CNS tissue. Gene therapy targeting the CNS has been tested in a number of studies and it was associated with little or no detectable immune response to the capsid (9, 16, 28, 34–37). While immune responses to AAV may be problematic, responses to the therapeutic transgene pose a greater risk for patients since they may inhibit the delivery of the therapeutic enzyme, as shown for lysosomal storage disease in pre-clinical models (11, 38, 39), and in humans (40–43). In the present study, we report that intracerebral gene therapy with rAAV2/5 encoding NAGLU in children with MPS IIIB syndrome triggers a peripheral cellular CD4^+^ and CD8^+^ T cell response to the therapeutic enzyme. This was assessed by measuring the proliferation of freshly isolated PBMCs exposed *ex-vivo* to rNAGLU or NAGLU peptide, and the expression of intracellular cytokines (IFN-γ, TNF-α, IL-2) by whole blood T cells exposed to the therapeutique enzyme. Cellular responses persisted during the 5.5 years of follow-up, but transiently, and they were still detected at M66 post-surgery suggesting the lack of acquired immunological tolerance. However, cellular immunity to the transgene did not prevent its long-term expression by brain cells, possibly a consequence of the long-term immunosuppressive treatment. Immunosuppression was started 14 days before surgery, and progressively reduced from 3 months to 30 months and then adjusted during the extension phase (from M30 to M66) to obtain trough blood concentrations of tacrolimus of 4 to 6 ng/ml.

NAGLU activity was not detected in any patient’s CSF and plasma at inclusion (20), and consistently we saw no CD4^+^ and CD8^+^ T cell responses to NAGLU. At M66 post-surgery, NAGLU activity was persistently detected in lumbar CSF (**Figure 3** and Deiva et al. submitted to publication), indicating the efficiency of vector delivery and gene transfer to the brain. Circulating T cells reactive to NAGLU were detectable rapidly after vector delivery, and appeared by waves throughout the follow-up. In these patients, the immune system perceives the AAV-derived transgene as non-self. Detection of vector genome in circulating blood for 2 days and transient detection of NAGLU in plasma for 1 month (20) suggest that particles deposited in brain tissue transited into the systemic circulation. Contact with peripheral dendritic cells or tissue macrophages during this period might account for antigenic presentation of NAGLU epitopes to lymphocytes. This systemic immune response may eliminate transgene expression and thus curtails the therapeutic efficacy of gene therapy (31). The mechanisms by which the immune system might abolish transgene expression from transduced brain cells are unclear and were documented in very few clinical trials. Activated CD8^+^ T cells release inflammatory mediators to promote immune responses (e.g., CCL3, TNF-α, and INF-γ) as well as cytotoxic molecules enabling direct cell killing (e.g., perforin and granzyme B) (44). Evidence of T cell-mediated anti-transgene cytotoxic T cell responses was documented in a phase 1/2 trial of intramuscular AAV-mediated gene transfer in Duchenne muscular dystrophy patients, where poor expression of dystrophin was associated with the detection of transgene-specific polyfunctional CD8 T cells (45). The induction of IFN-γ-expressing CD8 T cells to the therapeutic product was also reported in a α-1-antitrypsin (AAT)-deficient subject receiving AAV1-AAT treatment resulting in reduction in transgene expression (46). In our study, the priming of multifunctional CD4^+^ and CD8^+^ T cells reactive to NAGLU transgene did not prevent long-term transgene expression by brain cells. These findings raise the question of the positive impact of the immunosuppressive regimen. The rationale for including immunosuppression was based on our previous findings in dogs with MPS IIIB, which demonstrated the absolute requirement for immunosuppression to prevent neuroinflammation and elimination of transduced cells (11). Immunosuppression may prevent unwanted immune responses and promote functional tolerance through a combination of mechanisms including T-cell anergy, T-cell exhaustion by apoptosis and suppression by regulatory T cells (47–51). How these therapies may impact long-term immunity requires careful consideration, as long-term immunosuppression poses protracted risks, particularly in infants and children.

Neuroinflammatory responses after CNS-targeted delivery of AAV are a critical concern (28). Inflammation can be initiated within the CNS compartment, likely mediated by resident cells (e.g., microglia, astrocytes, oligodendrocytes), or from outside of the CNS and mediated by infiltrating peripheral T cells, breakdown of the blood–brain barrier, pro-inflammatory cytokines and other mechanisms (52). MRI did not detect oedema, inflammation, or signs of local necrosis at vector delivery sites in the four children up to 30 months after treatment (20), which is consistent with our findings in 25 dogs treated with a similar protocol and, for some of them, with the same material (11). Cytokines and chemokines in the CSF are markers of immune activation and they can also be used for tracking neuroinflammation (53). We assessed the concentration of 27 cytokines and chemokines in CSF from the four patients at inclusion. A status of neuroinflammation is suggested by the elevated concentrations of IFN-γ, IL-7, IL-9, IL-13 and IL-15, cytokines barely detectable in CSF from heathy children (29). Severe neuroinflammation is one of the hallmarks of MPS III in both patients and the mouse models (54). Activated microglia have been shown to express high levels of inflammatory cytokines and other proteins related to immunity and macrophage function. For example, a mouse model of MPS IIIB had increased brain transcript levels of the cytokine IFN-γ and its receptor (55), and another study demonstrated in this model upregulation of over 120 gene transcripts related to both innate and adaptive components of the immune system including microglia, macrophages, T cells, Toll-like receptors (TLR), and cytokines (56). Ausseil et al. have shown that heparan sulfate is a ligand of the TLR4 and mediates activation of microglia. In 10-day-old MPS IIIB NAGLU knockout mice, activated microglia and the levels of cytokine MIP1α were upregulated, suggesting that heparan sulfate primed microglia early in the course of the disease (57). During the first year post-surgery, CSF cytokines and chemokines concentrations remained stable, except for P3 who showed increased concentrations of IFN-γ and IP-10. Based on these results, we conclude that intracerebral gene therapy with rAAV2/5 encoding NAGLU did not increase neuroinflammation in three out of the four children with MPS IIIB syndrome.

After 5.5 years, safety and sustained enzyme production in the brain were observed in the 4 treated patients (Deiva et al. Submitted to publication). Cognitive benefit was not observed in the 3 oldest patients (Patients 2, 3 and 4), indicating that treatment had no or little impact on cognitive performances. In contrast, Patient 1 persistently acquired skills over the 66 months of follow-up, with higher performances as compared with the natural history of the disease. They were nevertheless not acquired at the same rate as normal children (Deiva et al. submitted to publication). Patient 1 had normal brain imaging with no sign of atrophy on the last MRI performed 66 months after surgery, in contrast to the 3 oldest patients (Deiva et al. submitted to publication). Strikingly, the immunological results shown herein demonstrate that the milder disease progression in patient 1 is associated with a very low level and less differentiated circulating NAGLU-specific CD4^+^ and CD8^+^ T cells, together with low concentrations of CSF cytokines and chemokines at baseline and during the follow-up.

To conclude, a detailed analysis of cellular immune responses elicited in rAAV2/5-NAGLU clinical trial clearly demonstrated the development of polyfunctional T cells specific for NAGLU transgene, detected as soon as 3 months post-surgery in all enrolled patients. This T cell response persisted over the 66 months of follow-up, but with no apparent impact on transgene expression, probably as a result of immunosuppressive regimen. However, we cannot exclude that the immune response may compromise the outcome of the therapy considering that the patient with milder disease progression had very low levels of NAGLU-reactive T cells and no sign of neuroinflammation post-surgery. This is the first comprehensive immunological analysis performed in intracerebral AAV-based gene therapy trials. A systematic and standardized immunomonitoring approach would be important for understanding the impact immune reactions can have on treatment safety and efficacy, helping the management of inflammatory responses with immunosuppression strategies, and identifying biomarkers to evaluate neuroinflammation before and after gene therapy.

## Data Availability Statement

The raw data supporting the conclusions of this article will be made available by the authors, without undue reservation.

## Ethics Statement

The studies involving human participants were reviewed and approved by Trial registration: EudraCT, number 2012-000856-33; International standard clinical trial registry, number ISRCTN19853672; Clinical trial.gov, NCT03300453. Written informed consent to participate in this study was provided by the participants’ legal guardian/next of kin.

## Author Contributions

M-LG, BP-B, JA, MZ, CA, J-MH, KD, and MT conceived the study. M-LG, BP-B, and JA performed the experiments. M-LG, BP-B, and JA analyzed the data. M-LG, BP-B, JA, MZ, CA, J-MH, KD, and MT drafted the manuscript. M-LG, BP-B, JA, MZ, CA, J-MH, KD, and MT revised and approved the manuscript. All authors contributed to the article and approved the submitted version.

## Funding

Institut Pasteur sponsored the study during the first 30 months and UniQure sponsored it for the following 36 months. The study was supported by funds from the “*Association Française Contre les Myopathies (AFM)*”, the patients ‘association “*Vaincre les Maladies Lysosomales*”, the Institut Pasteur, the Conny Maeva Charitable Foundation, and a gift from the Akbaraly family. The sponsors had no involvement in the collect, interpretation of data and in the writing of the report.

## Conflict of Interest

MT has received consulting fees from UniQure.

The remaining authors declare that the research was conducted in the absence of any commercial or financial relationships that could be construed as a potential conflict of interest.
